# Complications from percutaneous-left ventricular assist devices versus intra-aortic balloon pump in acute myocardial infarction-cardiogenic shock

**DOI:** 10.1371/journal.pone.0238046

**Published:** 2020-08-24

**Authors:** Saraschandra Vallabhajosyula, Anna V. Subramaniam, Dennis H. Murphree, Sri Harsha Patlolla, Lina Ya’Qoub, Vinayak Kumar, Dhiran Verghese, Wisit Cheungpasitporn, Jacob C. Jentzer, Gurpreet S. Sandhu, Rajiv Gulati, Nilay D. Shah, Bernard J. Gersh, David R. Holmes, Malcolm R. Bell, Gregory W. Barsness

**Affiliations:** 1 Department of Cardiovascular Medicine, Mayo Clinic, Rochester, Minnesota, United States of America; 2 Division of Pulmonary and Critical Care Medicine, Department of Medicine, Mayo Clinic, Rochester, Minnesota, United States of America; 3 Center for Clinical and Translational Science, Mayo Clinic Graduate School of Biomedical Sciences, Rochester, Minnesota, United States of America; 4 Section of Interventional Cardiology, Division of Cardiovascular Medicine, Department of Medicine, Emory University School of Medicine, Atlanta, Georgia, United States of America; 5 Department of Medicine, Mayo Clinic, Rochester, Minnesota, United States of America; 6 Department of Health Sciences Research, Mayo Clinic, Rochester, Minnesota, United States of America; 7 Department of Cardiovascular Surgery, Mayo Clinic, Rochester, Minnesota, United States of America; 8 Division of Cardiovascular Medicine, Department of Medicine, Louisiana State University School of Medicine, Shreveport, Louisiana, United States of America; 9 Department of Medicine, Amita Health Saint Joseph Hospital, Chicago, Illinois, United States of America; 10 Division of Nephrology, Department of Medicine, University of Mississippi School of Medicine, Jackson, Mississippi, United States of America; 11 Robert D. and Patricia E. Kern Center for the Science of Healthcare Delivery, Mayo Clinic, Rochester, Minnesota, United States of America; Erasmus Medical Center, NETHERLANDS

## Abstract

**Background:**

There are limited data on the complications with a percutaneous left ventricular assist device (pLVAD) vs. intra-aortic balloon pump (IABP) in acute myocardial infarction-cardiogenic shock (AMI-CS).

**Objective:**

To assess the trends, rates and predictors of complications.

**Methods:**

Using a 17-year AMI-CS population from the National Inpatient Sample, AMI-CS admissions receiving pLVAD and IABP support were evaluated for vascular, lower limb amputation, hematologic, neurologic and acute kidney injury (AKI) complications. In-hospital mortality, hospitalization costs and length of stay in pLVAD and IABP cohorts with complications was studied.

**Results:**

Of 168,645 admissions, 7,855 (4.7%) receiving pLVAD support. The pLVAD cohort had higher comorbidity, cardiac arrest (36.1% vs. 29.7%) and non-cardiac organ failure (74.7% vs. 56.9%) rates. Complications were higher in pLVAD compared to IABP cohort–overall 69.0% vs. 54.7%; vascular 3.8% vs. 2.1%; lower limb amputation 0.3% vs. 0.3%; hematologic 36.0% vs. 27.7%; neurologic 4.9% vs. 3.5% and AKI 55.4% vs. 39.1% (all *p*<0.001 except for amputation). Non-White race, higher comorbidity, organ failure, and extracorporeal membrane oxygen use were predictors of complications for both cohorts. The pLVAD cohort with complications had higher in-hospital mortality (45.5% vs. 33.1%; adjusted odds ratio 1.65 [95% confidence interval 1.55–1.75]), shorter duration of hospital stay, and higher hospitalization costs compared to the IABP cohort with complications (all *p*<0.001). These results were consistent in propensity-matched pairs.

**Conclusions:**

AMI-CS admissions receiving pLVAD had higher rates of complications compared to the IABP, with worse in-hospital outcomes in the cohort with complications.

## Introduction

Cardiogenic shock (CS) is a critical state of end-organ hypoperfusion caused by primary cardiac disease, with up to 80% caused by acute myocardial infarction (AMI) [1]. In addition to standard therapies for AMI-CS including reperfusion and vasoactive medications, mechanical circulatory support (MCS) devices are often employed to decrease myocardial oxygen demand, reduce left ventricular wall stress, and thus aid in myocardial recovery [1–3]. The intra-aortic balloon pump (IABP) was the first MCS device introduced in the 1960s, and has remained the device of choice in AMI-CS until recently [4]. However, large-scale randomized trials like IABP-SHOCK II (Intra-aortic balloon counterpulsation in acute myocardial infarction complicated by cardiogenic shock) have demonstrated no difference in AMI-CS outcomes with or without the IABP [5]. Newer devices including TandemHeart, Impella and extracorporeal membrane oxygenation (ECMO) have been introduced into clinical practice in the last 10–15 years, with Impella being the most commonly used today [6–17].

The Impella device provides significantly more hemodynamic support than IABP, with increases of 2.5 to 5 L/minute in cardiac output, however has yielded similar clinical outcomes in AMI-CS when compared with the IABP [1, 3, 18]. The PROTECT II study (Prospective, Randomized Clinical Trial of Hemodynamic Support with Impella 2.5 Versus Intra-Aortic Balloon Pump) showed similar outcomes between the Impella 2.5 and IABP in patients high-risk percutaneous coronary intervention (PCI), however it was underpowered to detect differences in complications [18]. There are limited data on the complications in patients using a percutaneous left ventricular assist device (pLVAD), such as an Impella or a TandemHeart and the IABP [6, 7, 18–21]. The few available studies investigating complications of pLVAD have been in small cohorts and are underpowered to detect differences in complication rates, therefore we sought to address this knowledge gap in a larger, nationally-representative sample of patients with AMI-CS [22, 23].

Using a nationally-representative hospital database, we sought to assess the contemporary national rates, temporal trends, and clinical outcomes of complications with the use of pLVAD compared to IABP. We hypothesized that during this 12-year study period, the complications from the pLVAD would decrease due to greater familiarity with the insertion and management of this device. We also sought to explore the predictors of complications and outcomes of the admissions experiencing these complications.

## Material and methods

Institutional Review Board approval was not sought for this study due to the publicly available nature of the de-identified data. The National (Nationwide) Inpatient Sample (NIS) is the largest all-payer database of hospital inpatient stays in the United States. NIS contains discharge data from a 20% stratified sample of community hospitals and is a part of the Healthcare Cost and Utilization Project (HCUP), sponsored by the Agency for Healthcare Research and Quality [24]. Information regarding each discharge includes patient demographics, primary payer, hospital characteristics, principal diagnosis, up to 24 secondary diagnoses, and procedural diagnoses. These data are available to other authors via the HCUP-NIS database with the Agency for Healthcare Research and Quality. Using the HCUP-NIS data from 2005–2016, a retrospective cohort study of admissions with AMI in the primary diagnosis field (International Classification of Diseases 9.0 Clinical Modification [ICD-9CM] 410.x and ICD-10CM I21.x-22.x) and a secondary diagnosis of CS (ICD-9CM 785.51, ICD-10CM R57.0) were identified [25]. Use of pLVAD (ICD-9CM 37.68; ICD-10PCS 5A0211D, 5A0221D, 02HA3RJ, 02HA4RJ) and IABP (ICD-9CM 37.61; ICD-10PCS 5A02110, 5A02210) was identified for all admissions consistent with prior literature [7, 12–14, 17, 26]. Since International Classification of Diseases 9 Clinical Modification (ICD-9CM) codes were re-defined in 2005 to distinguish the durable LVAD from short-term non-implantable devices or para-corporeal devices, admissions before 2005 were excluded from this study [7, 13, 14]. The administrative coding for pLVAD identifies both Impella and TandemHeart and does not distinguish between the various types of Impella devices (2.5, CP and 5.0). We also excluded AMI-CS admissions without MCS use, those receiving both a pLVAD and an IABP during the same admission and admissions without in-hospital mortality data. Similar to prior literature from the HCUP-NIS, we used the procedure day for pLVAD or IABP implantation to time placement. The Deyo’s modification of the Charlson Comorbidity Index was used to identify the burden of co-morbid diseases (**S1 Table**) [27]. Demographic characteristics, hospital characteristics, acute organ failure, coronary angiography, PCI and non-cardiac organ support use were identified for all admissions using previously used methodologies from our group [7, 12–14, 17, 26, 28–32].

Similar to prior literature, we identified relevant complications and categorized them as–(a) vascular complications–arterial injury, acquired arterio-venous fistula, vascular complications requiring surgery; (b) lower limb amputation; (c) hematologic–post-operative hemorrhage, hemolytic anemia, thrombocytopenia, blood transfusion; (d) neurologic–ischemic or hemorrhagic stroke; and (e) acute kidney injury (AKI) (**S1 Table**) [7, 33–36]. We did not include critical limb ischemia under vascular complications since there is no reliable way to distinguish acute from chronic limb ischemia using administrative codes [37, 38]. The primary outcome was the rates of complications in admissions receiving pLVAD compared to the IABP. Secondary outcomes included temporal trends, predictors, in-hospital mortality, hospitalization costs and hospital length of stay for admissions with complications in the IABP and pLVAD cohorts.

### Statistical analysis

As recommended by HCUP-NIS, survey procedures using discharge weights provided with HCUP-NIS database were used to generate national estimates. Using the trend weights provided by the HCUP-NIS, samples from 2005–2011 were re-weighted to adjust for the 2012 HCUP-NIS re-design [39]. All analyses were conducted accounting for clustering of admissions within a hospital (HOSP_NIS), weighting (DISCWT), and stratification (NIS_STRATUM) of the NIS consistent with prior data [12]. One-way analysis of variance and t-tests were used to compare categorical and continuous variables respectively. Logistic regression was used to analyze trends over time (referent year 2005). The inherent restrictions of the HCUP-NIS database related to research design, data interpretation, and data analysis were reviewed and addressed [39]. Pertinent considerations include not assessing individual hospital-level volumes (due to changes to sampling design detailed above), treating each entry as an ‘admission’ as opposed to individual patients, restricting the study details to inpatient factors since the HCUP-NIS does not include outpatient data, and limiting administrative codes to those previously validated and used for similar studies. Univariable analysis for trends, predictors and outcomes was performed and were represented as odds ratio (OR) with 95% confidence interval (CI). Multivariable logistic regression analysis incorporating age, sex, race, primary payer status, year of admission, hospital characteristics, comorbidities, acute organ failure, cardiac arrest, AMI type, cardiac procedures, extracorporeal membrane oxygenation support (ECMO) use, and non-cardiac procedures was performed for predictors of complications and in-hospital mortality. For the multivariable modeling, regression analysis with purposeful selection of statistically (liberal threshold of *p*<0.20 in univariate analysis) and clinically relevant variables was conducted. *A priori* sensitivity analyses were performed comparing the occurrence of complications and in-hospital mortality in admissions with complications stratified by type of AMI-CS, receipt of concomitant or subsequent ECMO, cardiac arrest, early (day zero) vs. delayed pLVAD/IABP placement, and in those undergoing cardiac surgery during the same admission.

Additionally, we performed a propensity-matched analysis. Age, sex, primary payer, race, Charlson comorbidity index, hospital region, hospital teaching status and location, hospital bedsize, acute respiratory failure, acute hepatic failure, acute neurological failure, cardiac arrest, type of AMI-CS, use of coronary angiography, PCI, invasive hemodynamic monitoring, ECMO, mechanical ventilation and acute hemodialysis were used as covariates in multivariable logistic regression model to develop the propensity-matched pairs. For the propensity matching, all variables had <1% missing variables. Using 1:1 nearest neighbor matching, 1,419 matching pairs (2,838 individual admissions) were developed for further use. The propensity-matched sample had standardized differences <10% for all baseline characteristics. The c-statistic for the propensity-score model was 0.89, suggestive of a good fit. The McNemar chi-square test and paired sample t-tests were used to compare categorical and continuous variables respectively in the propensity-matched sample. Two-tailed *p*<0.05 was considered statistically significant. All statistical analyses were performed using SPSS v25.0 (IBM Corp, Armonk NY).

## Results

### Baseline characteristics and temporal trends

**Fig 1:**
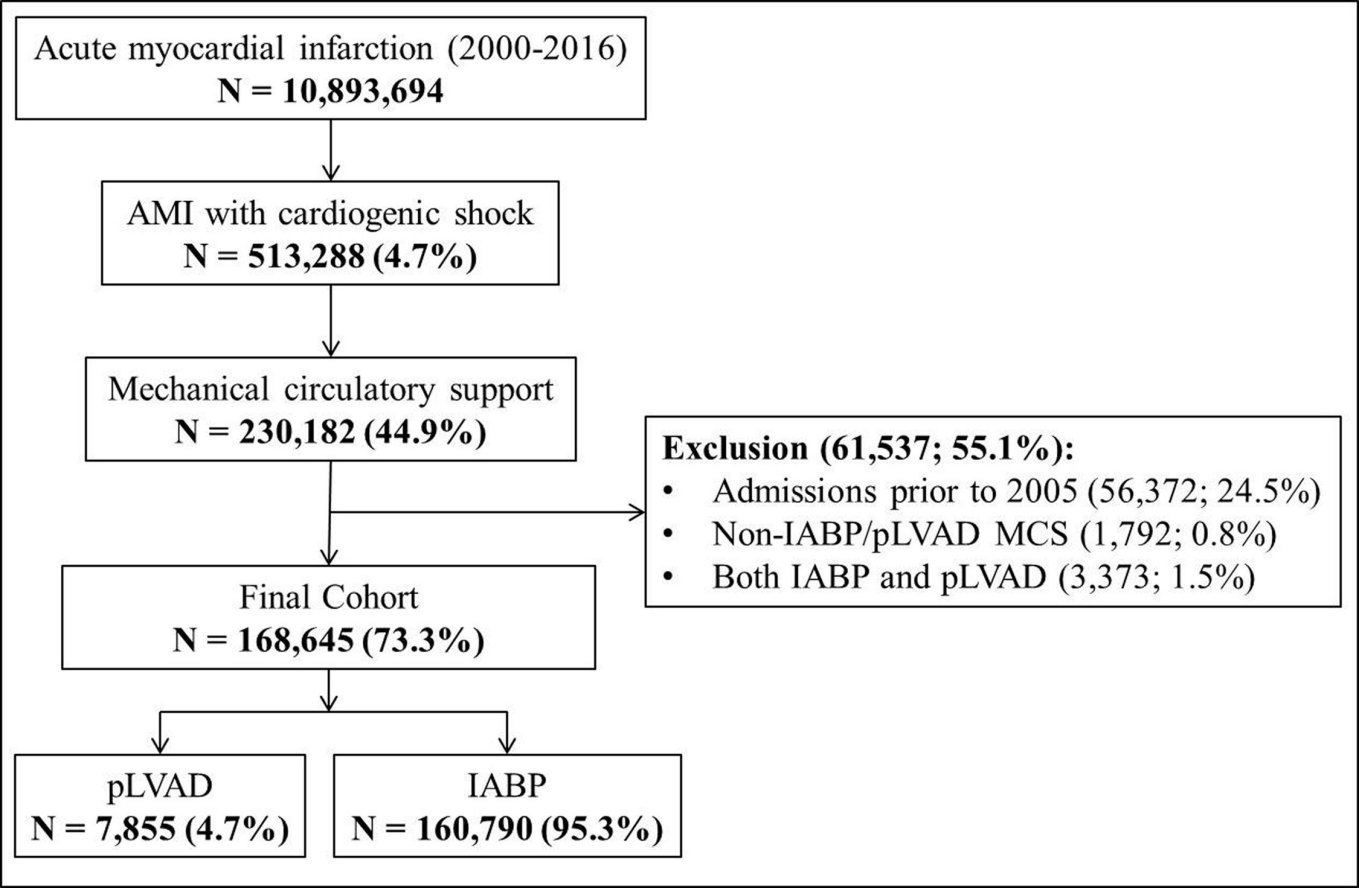
Consort diagram for selection of study cohort. Abbreviations: AMI: acute myocardial infarction; IABP: intra-aortic balloon pump; MCS: mechanical circulatory support; pLVAD: percutaneous left ventricular assist device.

**Fig 2:**
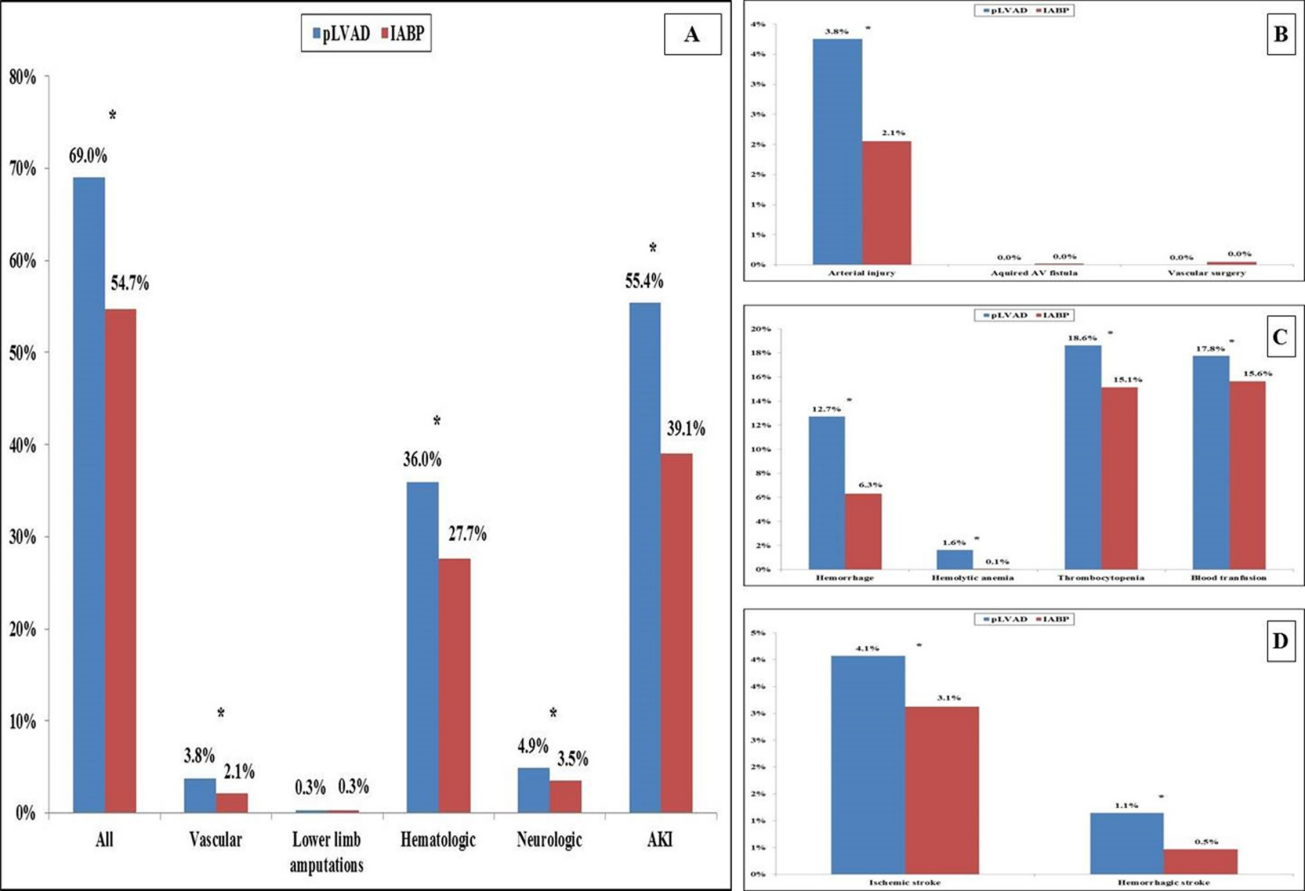
Complications in admissions receiving pLVAD and IABP support for AMI-CS. Legend: Cumulative complication rates (A) and individual components of vascular complications (B), hematologic complications (C) and neurologic complications (D); *p<0.05. Abbreviations: AKI: acute kidney injury; AV: arterio-venous; IABP: intra-aortic balloon pump; pLVAD: percutaneous left ventricular assist device.

**Fig 3:**
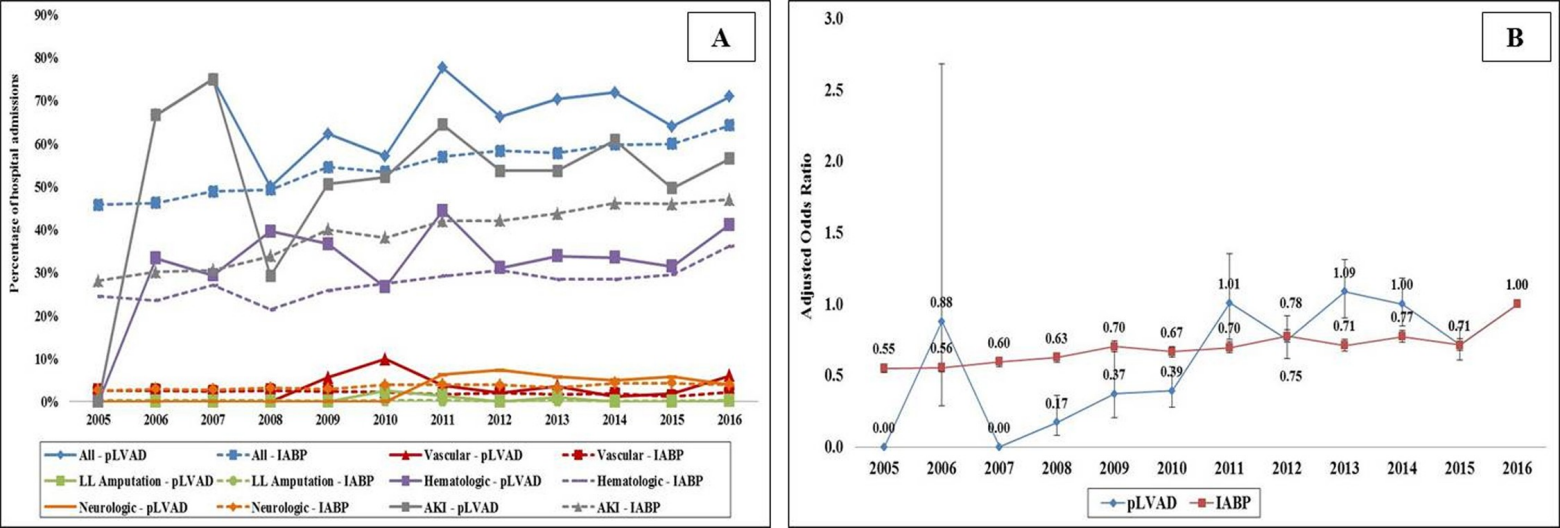
Trends of complications in AMI-CS supported with pLVAD and IABP. Legend: A: Unadjusted temporal trends in AMI-CS by pLVAD and IABP use (*p*<0.001 for trend over time); D: Adjusted multivariate logistic regression temporal trends of complications stratified by pLVAD and IABP use with 2016 as referent year; adjusted for age, sex, race, comorbidity, primary payer, socio-economic stratum, hospital characteristics, AMI type, acute organ failure, cardiac arrest, coronary angiography, percutaneous coronary intervention, invasive hemodynamic monitoring; extracorporeal membrane oxygenation use, invasive mechanical ventilation, hemodialysis and receipt of cardiac surgery (*p*<0.001 for trend over time). Abbreviations: AKI: acute kidney injury; IABP: intra-aortic balloon pump; LL: lower limb; pLVAD: percutaneous left ventricular assist device.

In the period between January 1, 2005 and December 31, 2016, 168,645 met our inclusion criteria (**Fig 1**). The pLVAD was used in 7,855 (4.7%) of the studied admissions. Compared to those receiving IABP, the cohort receiving pLVAD support was on average younger, of male sex, White race, with higher comorbidity and severity of illness, and more often experienced concomitant cardiac arrest (**Table 1**). Complications were noted in 93,317 (55.3%) admissions, with the cohort receiving pLVAD having higher cumulative rates of complications (69.0% vs. 54.7%; *p*<0.001). Arterial injury, hemorrhage, thrombocytopenia, blood transfusions, stroke and AKI were more common in the pLVAD cohort compared to IABP (**Fig 2**). The 12-year unadjusted and adjusted temporal trends of complications demonstrated a temporal increase in complication prevalence across both device categories (**Fig 3**). The pLVAD group persistently had higher complication rates than the IABP group after adjustment for patient and hospital characteristics.

**Table 1:** Baseline characteristics of AMI-CS admissions receiving pLVAD and IABP.

Characteristic		Total cohort			Propensity-matched cohort		
		pLVAD (N = 7,855)	IABP (N = 160,790)	*P*	pLVAD (N = 1,419)	IABP (N = 1,419)	*P*
**Age (years)**		64.8±12.1	66.0±12.3	<0.001	64.7±12.2	64.4±12.8	0.48
**Female sex**		28.3	32.0	<0.001	28.5	28.3	0.48
**Race**	**White**	66.3	64.6	0.002	64.1	61.4	0.14
	**Non-White**^**a**^	33.7	35.4		35.9	38.6	
**Primary payer**	**Medicare**	51.4	52.7	0.04	51.6	48.6	0.29
	**Medicaid**	8.7	8.1		8.7	10.9	
	**Private**	29.9	28.8		29.9	31.4	
	**Others**^**b**^	9.9	10.3		9.9	9.2	
**Quartile of median household income for zip code**	**0-25**^**th**^	31.7	26.6	<0.001	24.4	23.0	0.30
	**26**^**th**^**-50**^**th**^	28.1	26.6		23.0	24.7	
	**51**^**st**^**-75**^**th**^	22.1	25.0		24.1	23.5	
	**75**^**th**^**-100**^**th**^	18.1	21.8		21.4	19.8	
**Hospital teaching status and location**	**Rural**	3.0	4.7	<0.001	3.4	3.0	0.81
	**Urban non-teaching**	23.9	37.0		24.9	25.0	
	**Urban teaching**	73.1	58.3		71.7	72.0	
**Hospital bed-size**	**Small**	7.4	7.5	0.01	7.5	7.8	0.75
	**Medium**	23.5	22.1		23.5	22.3	
	**Large**	69.1	70.4		69.1	69.8	
**Hospital region**	**Northeast**	15.0	17.3	<0.001	15.9	15.0	0.69
	**Midwest**	18.2	24.4		15.7	20.4	
	**South**	46.1	37.5		47.5	39.5	
	**West**	20.7	20.8		20.9	25.2	
**Charlson Comorbidity Index**	**0–3**	31.6	30.5	<0.001	31.1	32.4	0.51
	**4–6**	46.1	52.0		46.5	44.3	
	**≥ 7**	22.3	17.5		22.3	23.3	
**AMI type**	**STEMI-CS**	64.5	70.9	<0.001	64.5	64.8	0.91
	**NSTEMI-CS**	35.5	29.1		35.5	35.2	
**Acute organ failure**	**Respiratory**	69.6	50.6	<0.001	69.1	67.4	0.18
	**Hepatic**	21.5	11.1	<0.001	21.2	22.2	0.55
	**Neurologic**	20.2	16.2	<0.001	20.0	20.2	0.96
**Cardiac arrest**		36.1	29.7	<0.001	35.2	35.2	>0.99
**Coronary angiography**		91.5	90.9	0.05	91.9	92.0	>0.99
**Percutaneous coronary intervention**		76.7	65.6	<0.001	77.0	75.4	0.36
**Coronary artery bypass grafting**		9.2	26.9	<0.001	—	—	—
**Invasive hemodynamic monitoring**^**c**^		30.9	23.8	<0.001	30.0	29.8	0.94
**Invasive mechanical ventilation**		53.0	45.4	<0.001	52.9	51.3	0.41
**Hemodialysis**		6.0	3.7	<0.001	6.3	6.8	0.65
**Cardiac surgery**		11.2	27.8	<0.001	—	—	—

**Legend:**Represented as percentage or mean ± standard deviation;

^a^Black, Hispanic, Asian, Native American, Others;

^b^Uninsured, No Charge, Others;

^c^pulmonary artery catheterization or right heart catheterization.

**Abbreviations:** AMI: acute myocardial infarction; CS: cardiogenic shock; IABP: intra-aortic balloon pump; NSTEMI: non-ST-segment elevation myocardial infarction; pLVAD: percutaneous left ventricular assist device; STEMI: ST-segment elevation myocardial infarction.

### Predictors of complications

In a multivariable logistic regression analysis, non-White race, higher comorbidity, admission to a large urban center, presence of acute organ failure, and utilization of ECMO support were predictors of complications in admissions receiving either pLVAD or IABP (**Table 2**). Advanced age, non-ST-segment elevation AMI-CS presentation, and invasive mechanical ventilation were predictors of complications in the IABP but not the pLVAD cohort (**Table 2**). In 2,838 propensity-matched pairs, the rates of complications were higher in the pLVAD cohort (70.9% vs. 63.5%; *p*<0.001).

**Table 2:** Predictors of complications in AMI-CS admissions receiving pLVAD and IABP.

Characteristic		pLVAD (N = 7,855)				IABP (N = 160,790)			
		Odds ratio	95% CI		*P*	Odds ratio	95% CI		*P*
			LL	UL			LL	UL	
**Age groups (years)**	**≤75 years**	Reference category				Reference category			
	**>75 years**	0.91	0.78	1.06	0.23	1.05	1.02	1.08	0.002
**Sex**	**Male**	Reference category				Reference category			
	**Female**	0.90	0.80	1.02	0.10	0.97	0.94	0.99	0.004
**Race**	**White**	Reference category				Reference category			
	**Non-White**^**a**^	1.17	1.04	1.32	0.008	1.10	1.07	1.12	<0.001
**Primary payer**	**Medicare**	Reference category				Reference category			
	**Medicaid**	1.00	0.81	1.23	0.99	1.06	1.02	1.11	0.008
	**Others**^**b**^	0.99	0.87	1.13	0.89	1.00	0.97	1.03	0.97
**Charlson Comorbidity Index**	**0–3**	Reference category				Reference category			
	**4–6**	2.09	1.83	2.39	<0.001	1.97	1.91	2.02	<0.001
	**≥ 7**	3.45	2.86	4.17	<0.001	3.99	3.83	4.15	<0.001
**Hospital teaching status and location**	**Rural**	Reference category				Reference category			
	**Urban non-teaching**	1.27	0.93	1.72	0.13	1.27	1.21	1.34	<0.001
	**Urban teaching**	1.57	1.16	2.11	0.003	1.55	1.48	1.64	<0.001
**Hospital bed-size**	**Small**	Reference category				Reference category			
	**Medium**	1.38	1.11	1.72	0.004	1.05	1.00	1.10	0.04
	**Large**	1.48	1.22	1.81	<0.001	1.14	1.09	1.19	<0.001
**Hospital region**	**Northeast**	Reference category				Reference category			
	**Midwest**	1.19	0.98	1.44	0.08	1.02	0.99	1.06	0.24
	**South**	1.05	0.89	1.24	0.55	1.11	1.08	1.15	<0.001
	**West**	0.85	0.70	1.03	0.09	1.13	1.09	1.18	<0.001
**AMI type**	**STEMI-CS**	Reference category				Reference category			
	**NSTEMI-CS**	0.96	0.85	1.09	0.56	1.35	1.31	1.38	<0.001
**Acute organ dysfunction**	**Respiratory**	1.96	1.73	2.23	<0.001	1.51	1.48	1.55	<0.001
	**Hepatic**	6.91	5.64	8.47	<0.001	4.40	4.21	4.60	<0.001
	**Neurologic**	2.31	1.97	2.70	<0.001	1.82	1.76	1.88	<0.001
**Cardiac arrest**		0.63	0.56	0.70	<0.001	0.83	0.81	0.85	<0.001
**Coronary angiography**		0.57	0.45	0.71	<0.001	0.80	0.77	0.83	<0.001
**Percutaneous coronary intervention**		0.72	0.63	0.83	<0.001	0.60	0.59	0.62	<0.001
**Invasive hemodynamic monitoring**^**c**^		1.69	1.49	1.91	<0.001	1.22	1.19	1.25	<0.001
**ECMO use**		1.73	1.23	2.41	0.001	2.22	1.95	2.53	<0.001
**Invasive mechanical ventilation**		1.01	0.89	1.14	0.92	1.48	1.45	1.52	<0.001

**Legend:** ^a^Black, Hispanic, Asian, Native American, Others;

^b^Uninsured, No Charge, Others;

^c^pulmonary artery catheterization or right heart catheterization.

**Abbreviations:** AMI: acute myocardial infarction; CI: confidence interval; CS: cardiogenic shock; ECMO: extracorporeal membrane oxygenation; IABP: intra-aortic balloon pump; LL: lower limit; NSTEMI: non-ST-segment elevation myocardial infarction; pLVAD: percutaneous left ventricular assist device; STEMI: ST-segment elevation myocardial infarction; UL: upper limit.

### In-hospital outcomes

The all-cause mortality was higher in the pLVAD cohort compared to the IABP cohort (45.0% vs. 29.5%; unadjusted OR 1.95 [95% CO 1.87–2.05]; *p*<0.001) in the overall cohort. The pLVAD cohort with complications had higher in-hospital mortality compared to the IABP cohort with complications (45.5% vs. 33.1%; unadjusted OR 1.68 [95% CI 1.59–1.78]; adjusted OR 1.65 [95% CI 1.55–1.75]; *p*<0.001) (**S2 Table**). pLVAD admissions that experienced complications were transferred to other hospitals more often (14.6% vs. 10.4%), had a shorter duration of hospital stay (12.6±15.1 vs. 13.5±12.9 days) and incurred higher hospitalization costs ($375,629±383,914 vs. 236,654±217,805) compared to the IABP admissions with complications (all *p*<0.001).

### Propensity-matched and sensitivity analyses

Propensity-matched cohorts had comparable baseline characteristics (**Table 1**). The all-cause in-hospital mortality was comparable in the propensity-matched cohort (25.4% vs. 25.3%, *p*>0.99). In 2,838 propensity-matched admissions, the cohort with complications had higher in-hospital mortality in the pLVAD group (28.4% vs. 26.7%; *p* = 0.04) compared to the IABP group.

**Fig 4:**
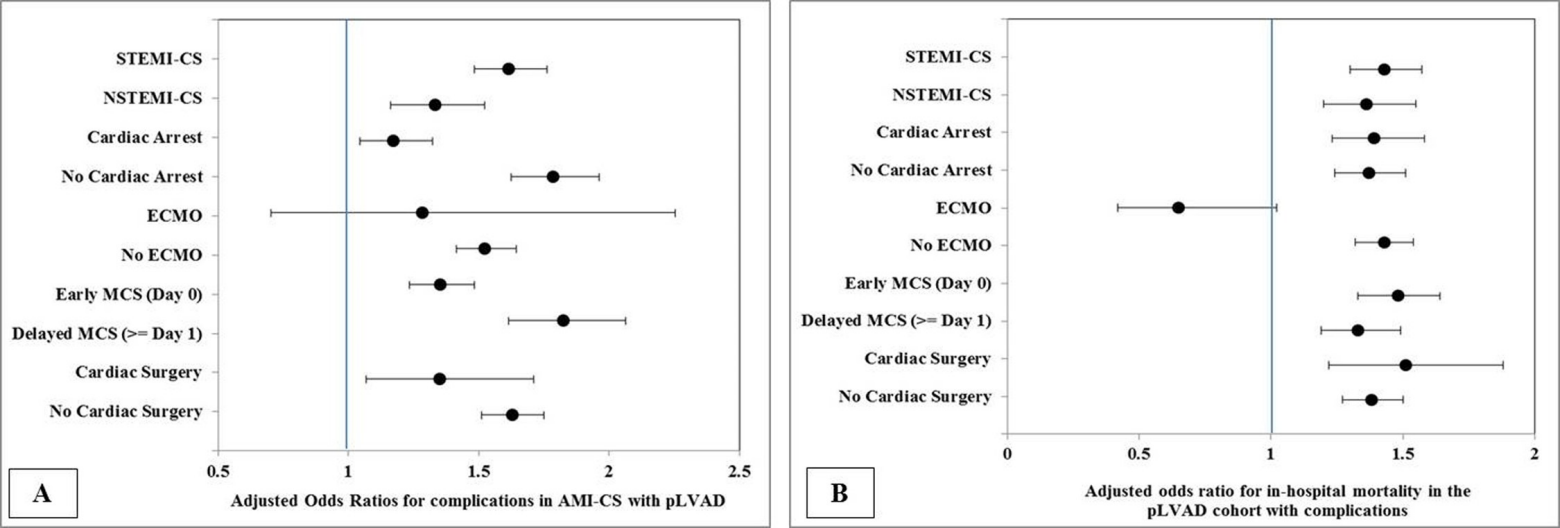
Multivariate predictors of complications and in-hospital mortality in the complications cohorts of AMI-CS supported with pLVAD compared to the IABP. Multivariable adjusted odds ratios (95% confidence intervals)* for occurrence of complications (A) and in-hospital mortality in admissions with complications in AMI-CS supported by pLVAD compared to IABP; all *p*<0.001 where 95% confidence interval does not include unity. *Adjusted for age, sex, race, comorbidity, primary payer, socio-economic stratum, hospital characteristics, AMI type, acute organ failure, cardiac arrest, coronary angiography, percutaneous coronary intervention, invasive hemodynamic monitoring; extracorporeal membrane oxygenation use, invasive mechanical ventilation, hemodialysis and receipt of cardiac surgery. Abbreviations: AMI: acute myocardial infarction; CS: cardiogenic shock; ECMO: extracorporeal membrane oxygenation; MCS: mechanical circulatory support; NSTEMI: non-ST-elevation myocardial infarction; STEMI: ST-elevation myocardial infarction.

In sensitivity analyses for burden of complications (**Fig 4A**) and in-hospital mortality (**Fig 4B**), AMI-CS admissions supported by pLVAD had higher adjusted odds for complications and higher adjusted odds for in-hospital mortality in the cohort with complications compared to the IABP cohort. Admissions that concomitantly or subsequently received ECMO support had comparable rates of complications and in-hospital mortality between the pLVAD and IABP cohorts (**Fig 4A and 4B**).

## Discussion

In a large nationally-representative study evaluating AMI-CS admissions receiving pLVAD or IABP for circulatory support, we noted that the cohort receiving pLVAD support was on average younger, with higher comorbidity, severity of illness and had greater rates of concomitant cardiac arrest. The pLVAD cohort had a higher rate of overall complications including vascular, hematologic, neurologic and AKI. There was a temporal increase in complication rates across both cohorts during this 12-year period. Higher comorbidity, greater illness severity, and use of ECMO support were predictors of complications in both pLVAD and IABP cohorts. The pLVAD cohort with complications had higher in-hospital mortality and greater resource utilization that was consistent across multiple relevant sub-groups.

This is one of the largest studies to holistically review and discuss complications of pLVAD and IABP use in AMI-CS. Prior studies from the HCUP-NIS database have discussed individual complications specifically, but have not investigated the broad categories of clinically relevant complications that are reviewed in the present study [37, 40, 41]. Our results are consistent with prior work demonstrating that pLVAD has a higher overall complication rate when compared to IABP [6, 20, 22, 23, 42]. In a cohort of 41 AMI-CS patients, Thiele et al. noted patients receiving a TandemHeart to have higher rates of severe bleeding and limb ischemia compared to the IABP [42]. Burkhoff et al. performed a similar randomized multicenter trial in 42 patients and found increased rate of adverse events in the TandemHeart group, but did not reach statistical significance [23]. In 2008, the ISAR-SHOCK (Efficacy Study of Left Ventricular Assist Device to Treat Patients with Cardiogenic Shock) trial compared the Impella LP 2.5 to IABP in 26 patients and did not note a difference in complication rates [22]. However it is important to note that patients who died during support were excluded from the outcomes analysis [22]. Finally, a recent meta-analysis of studies comparing Impella to IABP also found higher rates of complications, specifically sepsis and peripheral ischemia, in the Impella group [3]. In a recent study using the Premier Medical Database, Amin et al. noted the Impella to be associated with higher rates of adverse events and in-hospital mortality [6].

The pLVAD cohort in our real world study had higher comorbidity, higher rates of cardiac arrest and greater acuity of illness despite being younger, and developed higher rates of complications despite adjustment for these baseline risk factors and illness severity. It is possible that these disparities exist because pLVAD provides a higher level of hemodynamic support is thus chosen more often in severely ill and comorbid patient populations [1]. Though our study has attempted to control for potential confounders by use of multivariable regression analysis, we cannot eliminate the possibility of confounding by indication and the presence of unmeasured confounders. Furthermore, information regarding extent of coronary disease, success of revascularization and outcome of revascularization, hemodynamic management protocols and treatment-limiting decisions are not available in our database, which could also be factors affecting the choice of pLVAD or IABP. Expectedly the cohort with complications had higher in-hospital mortality, independent of the device used. However, the cohort with complications was transferred to other institutions more often, likely due to the need for sub-specialty medical or surgical expertise, include the need to upgrade to ECMO support. However, since the HCUP-NIS database does not track patients across hospital admissions, this hypothesis will need additional studies for confirmation. We have previously shown that complications are associated with higher in-hospital resource utilization and therefore judicious patient selection is key to employ these devices [36].

Several predictors of complications in AMI-CS admissions were identified including non-White race, higher comorbidity, admission to a large urban center, presence of acute organ failure, and utilization of ECMO support. These were present in both pLVAD and IABP admissions. Hepatic failure and ECMO support were strong predictors of worse outcomes. This is similar to prior studies of ECMO and Impella which found higher risk of bleeding requiring blood transfusion and development of acute kidney injury (AKI) [8]. This is potentially related to increased risk of hemolysis, vascular complications and hypovolemia causing pre-renal hypoperfusion [8]. In our study there were no unique predictors of complications in pLVAD, however, risk of complication in IABP was associated with advanced age, non-ST-segment elevation AMI-CS presentation and invasive mechanical ventilation. It is possible that given the significantly smaller pLVAD cohort, we were unable to identify unique predictors. Finally, our study demonstrates significant regional and hospital level variation in the risk of complications from IABP and pLVAD, consistent with prior work in AMI-CS and MCS [7, 13, 14, 43].

In our study, the pLVAD cohort with complications had higher in-hospital mortality compared to the IABP cohort with complications. Prior studies comparing pLVAD to IABP have either been underpowered to assess mortality [22, 23, 42], or have demonstrated no differences in in-hospital or 30-day mortality [3, 20]. This signal was consistent across the various pre-specified sub-groups when stratified by type of AMI-CS, presence of cardiac arrest, early vs. delayed MCS, and receipt of concomitant cardiac surgery. Our study adds to the current evidence of higher in-hospital mortality with pLVAD admissions with complications, and further investigation in prospective studies is needed to delineate patient-specific risk factors and guide clinical decision making [6]. The timing of MCS device insertion and the stage of CS that they might be useful in need further determination. The recognition of CS remains a clinical challenge that serves as a significant confounder in the care of these patients.

Lastly, this study showed a temporal increase in complication rates amongst both cohorts, contrary to the expected decrease in complication rates that should occur with increased operator experience over time. Given the administrative nature of the database, it is possible that these trends could be a function of systematic changes in coding over time similar to other literature published on this topic from the HCUP-NIS database [40, 41]. Further investigation is needed to determine patient-specific factors that may better delineate optimal candidates for pLVAD versus IABP and adherence to guideline-directed use of these MCS devices.

### Limitations

This study has several limitations, which are inherent to the analysis of a large administrative database. The HCUP-NIS attempts to mitigate potential errors by using internal and external quality control measures. The definition of CS was based on discharge diagnoses and not hemodynamic parameters. Information on vasoactive medication use and dosing, left ventricular function, peak serum lactate, and hemodynamic variables known to influence outcomes in this population, were unavailable in the HCUP-NIS database. The timing and duration of CS, which are known to influence mortality, could not be reliably measured from this database. Though the timing of pLVAD or IABP placement can be timed to the day of procedure, further granularity, including the timing of insertion relative to the PCI cannot be ascertained in this database. Further data are needed to assess the complications of the Impella device independently in comparison to the IABP. The lack of angiographic data, such target vessel for PCI, classification and the presence of multi-vessel disease, that may significantly influence outcomes, were not available in this database. Additionally, because of the non-randomized nature of this study it is challenging to fully understand the baseline differences in the groups and determine how this impacted on outcomes. Lastly, it is possible that our administrative codes capture pre-existing hemolytic anemia and thrombocytopenia in this population, as these codes cannot accurately distinguish acute from chronic for a given hospitalization. Despite these limitations, this study addresses an important knowledge gap highlighting the national trends and outcomes of in-hospital complications in AMI-CS receiving the pLVAD or IABP.

## Conclusions

In this study of AMI-CS admissions receiving pLVAD or IABP support, the pLVAD cohort had consistently higher rates of complications. Nearly 70% of all admissions receiving the pLVAD had complications in comparison to 55% of the IABP cohort. In this observational study, the cohort with pLVAD with complications had higher in-hospital mortality and resource utilization compared to the IABP cohort with complications, highlighting the need for further careful study in dedicated prospective studies.

## Supporting information

S1 Table Administrative codes used for diagnoses and procedures. (DOCX)

S2 Table Predictors of in-hospital mortality in AMI-CS with complications. (DOCX)

## Abbreviations

AKI: acute kidney injury
AMI: acute myocardial infarction
CI: confidence interval
CS: cardiogenic shock
ECMO: extracorporeal membrane oxygenation
HCUP: Healthcare Cost and Utilization Project
IABP: intra-aortic balloon pump
ICD-9CM: International Classification of Diseases-9 Clinical Modification
NIS: National/Nationwide Inpatient Sample
OR: odds ratio
PCI: percutaneous coronary intervention
pLVAD: percutaneous left ventricular assist device
