# Supplementary material for: Complications from percutaneous-left ventricular assist devices versus intra-aortic balloon pump in acute myocardial infarction-cardiogenic shock
Source: PLoS One. 2020 Aug 24;15(8):e0238046. doi: 10.1371/journal.pone.0238046 (PMC7444810; doi:10.1371/journal.pone.0238046)
Supplement: S1 Table — (DOCX) [file pone.0238046.s001.docx]

**S1 Table. Administrative codes used for diagnoses and procedures**

| **Comorbidity** | **ICD-9CM codes** |
| --- | --- |
| Cardiac arrest | 427.5 |
| Cardiogenic shock | 785.51 |
| Respiratory failure | 518.81, 518.82, 518.85, 786.09, 799.1, 96.7, 96.70, 96.71, 96.72 |
| Hepatic failure | 570.0, 572.2, 573.3, 573.4 |
| Hematologic failure | 286.6-286.9, 287.4, 287.5 |
| Neurological failure | 293, 293.0, 293.1, 293.8, 293.81-293.84, 293.89, 293.9, 348.1, 348.3, 348.30, 348.81, 348.39, 780.01, 780.09, 89.14 |
| Invasive hemodynamic assessment | 37.21, 37.23, 204 |
| Coronary angiography | 37.22, 37.23, 88.53-88.56 |
| Percutaneous coronary intervention | 00.66, 36.01, 36.02, 36.05, 36.06, 36.07, 88.57 |
| Extra-corporeal membrane oxygenation | 39.65 |
| Invasive mechanical ventilation | 96.7, 96.70, 96.71, 96.72 |
| Hemodialysis | 39.95 |
| Vascular complications | 904.0, 904.1, 904.2, 904.4, 904.40, 904.41, 904.7, 904.8, 904.9, 998.2, 999.2, 997.2, 997.7, 997.79 |
| Acquired AV fistula | 447.0 |
| Vascular complications requiring surgery | 39.31, 39.41, 39.49, 39.52, 39.53, 39.56, 39.57, 39.58, 39.59, 39.79 |
| Lower limb amputation | 84.1, 84.10, 84.11, 84.12, 84.13, 84.14, 84.15, 84.16, 84.17, 84.18, 84.19 |
| Post-operative hemorrhage/hematoma | 998.11, 998.12, 285.1 |
| Hemolytic anemia | 283.0, 283.1, 283.10, 283.11, 283.19, 283.2, 283.9 |
| Thrombocytopenia | 287.4, 287.41, 287.49, 287.5, 287.8, 287.9, 289.84 |
| Red blood cell transfusion | 99.00, 99.02, 99.03, 99.04 |
| Acute ischemic stroke | 433.01, 433.11, 433.21, 433.31, 433.81, 433.91, 436.0, 437.1, 434, 434.0, 434.00, 434.01, 434.1, 434.10, 434.11, 434.9, 434.90, 434.91, 435, 435.0, 435.1, 435.2, 435.3, 435.8, 435.9 |
| Intracranial hemorrhage | 430, 431, 432.0, 432.1, 432.9 |
| Acute kidney injury | 584, 584.5, 584.6, 584.7, 584.8, 584.9 |

**Abbreviations:** ICD-9CM: International Classification of Diseases, 9.0 Clinical Modifications
