# Supplementary material for: Complications from percutaneous-left ventricular assist devices versus intra-aortic balloon pump in acute myocardial infarction-cardiogenic shock
Source: PLoS One. 2020 Aug 24;15(8):e0238046. doi: 10.1371/journal.pone.0238046 (PMC7444810; doi:10.1371/journal.pone.0238046)
Supplement: S2 Table — (DOCX) [file pone.0238046.s002.docx]

**S2 Table. Predictors of in-hospital mortality in** **AMI-CS with complications**

| **Overall cohort**  **(N=168,645)** | | **Odds ratio** | **95% confidence interval** | | ***P*** |
| --- | --- | --- | --- | --- | --- |
|  |  |  | **Lower limit** | **Upper limit** |  |
| **pLVAD use** | | 1.65 | 1.55 | 1.75 | <0.001 |
| **Age groups (years)** | **≤75 years** | Reference category | | | |
|  | **>75 years** | 1.73 | 1.66 | 1.79 | <0.001 |
| **Sex** | **Male** | Reference category | | | |
|  | **Female** | 1.16 | 1.13 | 1.20 | <0.001 |
| **Race** | **White** | Reference category | | | |
|  | **Non-White^a^** | 1.04 | 1.01 | 1.07 | 0.01 |
| **Primary payer** | **Medicare** | Reference category | | | |
|  | **Medicaid** | 0.71 | 0.67 | 0.75 | <0.001 |
|  | **Others^b^** | 0.68 | 0.66 | 0.71 | <0.001 |
| **Charlson Comorbidity Index** | **0-3** | Reference category | | | |
|  | **4-6** | 1.10 | 1.06 | 1.15 | <0.001 |
|  | **≥ 7** | 1.08 | 1.02 | 1.13 | 0.006 |
| **Hospital teaching**  **status and location** | **Rural** | Reference category | | | |
|  | **Urban non-teaching** | 0.90 | 0.83 | 0.97 | 0.009 |
|  | **Urban teaching** | 0.96 | 0.88 | 1.04 | 0.28 |
| **Hospital bed-size** | **Small** | Reference category | | | |
|  | **Medium** | 1.08 | 1.01 | 1.15 | 0.02 |
|  | **Large** | 1.07 | 1.01 | 1.14 | 0.02 |
| **Hospital region** | **Northeast** | Reference category | | | |
|  | **Midwest** | 0.95 | 0.91 | 1.00 | 0.05 |
|  | **South** | 1.24 | 1.18 | 1.29 | <0.001 |
|  | **West** | 0.97 | 0.93 | 1.02 | 0.30 |
| **Year of admission** | **2005** | Reference category | | | |
|  | **2006** | 1.01 | 0.93 | 1.09 | 0.80 |
|  | **2007** | 0.86 | 0.80 | 0.93 | <0.001 |
|  | **2008** | 0.64 | 0.59 | 0.69 | <0.001 |
|  | **2009** | 0.65 | 0.60 | 0.70 | <0.001 |
|  | **2010** | 0.60 | 0.55 | 0.65 | <0.001 |
|  | **2011** | 0.56 | 0.52 | 0.61 | <0.001 |
|  | **2012** | 0.59 | 0.55 | 0.64 | <0.001 |
|  | **2013** | 0.60 | 0.56 | 0.65 | <0.001 |
|  | **2014** | 0.58 | 0.54 | 0.62 | <0.001 |
|  | **2015** | 0.52 | 0.48 | 0.57 | <0.001 |
|  | **2016** | 0.57 | 0.52 | 0.61 | <0.001 |
| **AMI type** | **STEMI-CS** | Reference category | | | |
|  | **NSTEMI-CS** | 0.77 | 0.75 | 0.80 | <0.001 |
| **Acute organ dysfunction** | **Respiratory** | 1.57 | 1.52 | 1.63 | <0.001 |
|  | **Hepatic** | 1.92 | 1.85 | 2.00 | <0.001 |
|  | **Neurologic** | 1.24 | 1.20 | 1.29 | <0.001 |
| **Cardiac arrest** | | 1.69 | 1.63 | 1.74 | <0.001 |
| **Coronary angiography** | | 0.78 | 0.75 | 0.82 | <0.001 |
| **Percutaneous coronary intervention** | | 1.05 | 1.02 | 1.08 | 0.003 |
| **Invasive hemodynamic monitoring^c^** | | 1.11 | 1.08 | 1.15 | <0.001 |
| **Extracorporeal membrane oxygenation use** | | 2.86 | 2.55 | 3.20 | <0.001 |
| **Invasive mechanical ventilation** | | 1.58 | 1.53 | 1.63 | <0.001 |
| **Hemodialysis use** | | 1.72 | 1.63 | 1.82 | <0.001 |

**Legend:** ^a^Black, Hispanic, Asian, Native American, Others; ^b^Uninsured, No Charge, Others; ^c^pulmonary artery/right heart catheter

**Abbreviations:** AMI: acute myocardial infarction; CS: cardiogenic shock; NSTEMI: non-ST-segment elevation myocardial infarction; pLVAD: percutaneous left ventricular assist device; STEMI: ST-segment elevation myocardial infarction
